# Increased plasma neutrophil gelatinase-associated lipocalin levels in poor-grade aneurysmal subarachnoid hemorrhage at admission to the ICU

**DOI:** 10.1186/cc9745

**Published:** 2011-03-11

**Authors:** M Terwiel, H De Geus, J Bakker, M Van der Jagt

**Affiliations:** 1 Erasmus MC, University Medical Center Rotterdam, the Netherlands

## Introduction

Neutrophil gelatinase-associated lipocalin (NGAL) and cystatin C (CyC) are powerful biomarkers predicting acute kidney injury (AKI) in the critically ill. In addition, both NGAL and CyC are related to systemic inflammation, cerebral ischemia and vascular wall damage. Aneurysmal subarachnoid hemorrhage (SAH) is frequently accompanied by cerebral ischemia and has been linked to systemic inflammation. We studied the relationship between NGAL and CyC levels and the severity grade of SAH at ICU admission.

## Methods

Thirty-six patients with SAH were recruited from a large prospective study on NGAL and AKI between September 2007 and April 2008. Patients with non-aneurysmal SAH (*n *= 3) and one patient with eGFR <60 ml/minute/1.73 m^2 ^were excluded. No subjects had AKI (RIFLE category Risk or more) or suffered from chronic kidney disease (CKD) stage 3 or more. We dichotomised patients into two groups: awake (GCS 15 to 11, *n *= 30) and comatose (GCS 10 to 3, *n *= 6), based on the Prognosis on Admission of Aneurysmal Subarachnoid Hemorrhage (PAASH) scale. Statistical comparisons were made with the Mann-Whitney U test and Spearman's rho test.

## Results

Plasma (p)NGAL was higher in comatose patients (median 144 ng/ml vs. 89 ng/ml, *P *< 0.05). No differences were found in urine NGAL plasma CyC and urine CyC levels or regular inflammatory parameters (leucocyte count, CRP and temperature). A confounding effect from mechanical ventilation on pNGAL production was excluded using the correlation statistics in intubated and non-intubated patients separately. After correction the correlation between GCS and pNGAL persisted in non-intubated patients (Spearman's rho (non-intubated, *n *= 29) -0.36, *P *< 0.05, and (intubated, *n *= 7) -0.62, *P *= 0.069). We found trends towards less positive fluid balance (*P *= 0.06) during the first 24 hours of admission and higher serum lactate (*P *= 0.08) in comatose patients, which did not reach statistical significance. Angiography-related contrast exposure was similar in both groups.

## Conclusions

Our results indicate that poor-grade SAH is associated with increased pNGAL levels at ICU admission not related to AKI, CKD or inflammatory parameters. Alternative mechanisms linking NGAL to SAH grade should therefore be investigated, such as increased sympathetic/catecholamine activity in poor-grade SAH patients [1].
